# LCRF‐0006, a small molecule mimetic of the N‐cadherin antagonist peptide ADH‐1, synergistically increases multiple myeloma response to bortezomib

**DOI:** 10.1096/fba.2019-00073

**Published:** 2020-06-15

**Authors:** Krzysztof M. Mrozik, Chee M. Cheong, Duncan R. Hewett, Jacqueline E. Noll, Khatora S. Opperman, Alaknanda Adwal, Darryl L. Russell, Orest W. Blaschuk, Kate Vandyke, Andrew C. W. Zannettino

**Affiliations:** ^1^ Myeloma Research Laboratory Adelaide Medical School Faculty of Health and Medical Sciences The University of Adelaide Adelaide Australia; ^2^ Precision Medicine Theme South Australian Health and Medical Research Institute (SAHMRI) Adelaide Australia; ^3^ Ovarian and Reproductive Cancer Biology Laboratory Robinson Research Institute The University of Adelaide Adelaide Australia; ^4^ Division of Urology Department of Surgery McGill University Montreal Canada; ^5^ Central Adelaide Local Health Network Adelaide Australia

**Keywords:** combination therapy, endothelial cell, multiple myeloma, tumor‐associated vasculature, vascular permeability

## Abstract

N‐cadherin is a homophilic cell‐cell adhesion molecule that plays a critical role in maintaining vascular stability and modulating endothelial barrier permeability. Pre‐clinical studies have shown that the N‐cadherin antagonist peptide, ADH‐1, increases the permeability of tumor‐associated vasculature thereby increasing anti‐cancer drug delivery to tumors and enhancing tumor response. Small molecule library screens have identified a novel compound, LCRF‐0006, that is a mimetic of the classical cadherin His‐Ala‐Val sequence‐containing region of ADH‐1. Here, we evaluated the vascular permeability‐enhancing and anti‐cancer properties of LCRF‐0006 using in vitro vascular disruption and cell apoptosis assays, and a well‐established pre‐clinical model (C57BL/KaLwRij/5TGM1) of the hematological cancer multiple myeloma (MM). We found that LCRF‐0006 disrupted endothelial cell junctions in a rapid, transient and reversible manner, and increased vascular permeability in vitro and at sites of MM tumor in vivo. Notably, LCRF‐0006 synergistically increased the in vivo anti‐MM tumor response to low‐dose bortezomib, a frontline anti‐MM agent, leading to regression of disease in 100% of mice. Moreover, LCRF‐0006 and bortezomib synergistically induced 5TGM1 MM tumor cell apoptosis in vitro. Our findings demonstrate the potential clinical utility of LCRF‐0006 to significantly increase bortezomib effectiveness and enhance the depth of tumor response in patients with MM.

**Abbreviations**
2‐HP‐β‐CD: 2‐hydroxypropyl‐β‐cyclodextrin
BLI: bioluminescence imaging
BMEC: bone marrow endothelial cell
CDI: coefficient of drug interaction
EC: endothelial cell
MM: multiple myeloma
MSC: mesenchymal stromal cell
OB: osteoblast
OP: osteoprogenitor

## 1 INTRODUCTION

The integrity and permeability of the endothelial barrier is tightly controlled and maintained through adhesive interactions between neighboring endothelial cells (ECs), as well as between ECs and adjacent mural cells (pericytes and smooth muscle cells).^1^, ^2^ In addition to molecules that mediate tight junctions between ECs (eg, occludin and claudins), cellular adhesion in blood vessels involves N‐cadherin and VE‐cadherin, which mediate calcium‐dependent, adherens junction‐type cell‐cell adhesion.^3^, ^4^, ^5^, ^6^ N‐cadherin is diffusely expressed on the EC surface and facilitates nascent EC‐EC junctions.^7^, ^8^, ^9^ In addition, N‐cadherin can co‐ordinate EC junction maturation by controlling VE‐cadherin expression.^8^, ^10^ N‐cadherin also plays a major role in the recruitment and adhesion of mural cells to the abluminal surface of ECs, thereby facilitating the remodeling, maturation and stabilization of vascular networks.^4^, ^11^

Tumor‐associated vasculature, essential to cancer cell growth, survival and metastasis, is widely recognized and investigated as a potential target for anti‐cancer therapy.^12^, ^13^ In comparison to normal vasculature, tumor‐associated vasculature is relatively immature and structurally abnormal, including defective EC junctions and loosely attached or absent pericytes, resulting in a weakened endothelial barrier.^14^, ^15^, ^16^, ^17^, ^18^ These characteristics are thought to render tumor‐associated vasculature more sensitive to vascular‐modulating agents, compared with normal vasculature.^19^, ^20^ The importance of N‐cadherin in vascular stability suggests that inhibition of N‐cadherin function is a potential mechanism to increase the porosity of tumor vasculature. In support of this, perturbation of N‐cadherin function has been shown to disrupt EC‐EC and EC‐mural cell junctions, and increase vascular permeability to molecules with high molecular mass both in vitro and in vivo.^21^, ^22^ For instance, treatment of confluent human umbilical vein‐derived EC monolayers with the N‐cadherin antagonist peptide ADH‐1 increased permeability to 40 kDa fluorescein isothiocyanate (FITC)‐conjugated dextran in vitro.^21^ In addition, ADH‐1 treatment in a rat melanoma xenograft model increased tumor delivery of molecules with high binding affinity for plasma proteins (eg, ~70 kDa albumin), including Evans blue dye and the chemotherapeutic agent melphalan.^21^ A Phase I/II clinical trial also found that ADH‐1 treatment, in combination with isolated limb intravenous infusion of melphalan improved initial response rates in patients with advanced melanoma, when compared with melphalan alone.^23^, ^24^ Notably, 60% of patients receiving the combination therapy achieved a partial response or greater, compared with 40% of patients receiving melphalan alone. However, the combination did not improve time to disease progression, compared with melphalan alone.^23^, ^24^ While preliminary, these data suggest that ADH‐1, and ADH‐1‐like agents, may also enhance tumor delivery of, and response to, anti‐cancer agents with high plasma protein binding affinity in other cancers.

**FIGURE 1:**
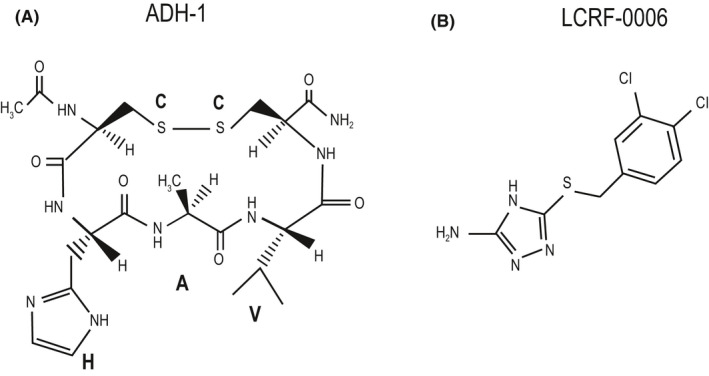
The structure of (A) the N‐cadherin antagonist peptide ADH‐1 and (B) the small molecule ADH‐1 mimetic LCRF‐0006

Synthetic small molecule mimetics of peptide drugs are thought to offer several advantages over their peptide counterparts, including enhanced proteolytic stability, bio‐availability and potency.^25^, ^26^ The small molecule LCRF‐0006 (compound number 35 in patent US 7,446,120 B2^27^) was identified as an ADH‐1 mimetic in a screen of compounds with three‐dimensional structures similar to the His‐Ala‐Val sequence‐containing region of ADH‐1 (Figure 1A,B). LCRF‐0006 was found to inhibit cerebellar neurite outgrowth on N‐cadherin‐expressing NIH‐3T3 cells in vitro, suggesting that LCRF‐0006 inhibits N‐cadherin‐dependent processes.^27^, ^28^

Multiple myeloma (MM) is a hematological cancer characterized by the clonal proliferation of malignant antibody‐producing plasma cells, and tumor‐associated angiogenesis, within the bone marrow.^29^, ^30^ In this study, we hypothesized that LCRF‐0006 would enhance the permeability of MM tumor‐associated vasculature to molecules with high binding affinity for plasma proteins and would increase MM tumor response to anti‐cancer therapy. To this end, we evaluated the vascular disruption and permeability‐enhancing properties of LCRF‐0006 in vitro and in vivo. In addition, we assessed the effectiveness of LCRF‐0006 in combination with a low dose of the plasma protein‐binding, frontline anti‐MM agent bortezomib (VELCADE^®^) in the well‐established, orthotopic C57BL/KaLwRij/5TGM1 mouse model of MM.

## 2 MATERIALS AND METHODS

### 2.1 Cell culture

Cell culture reagents were sourced from Merck, unless otherwise specified. All media were supplemented with 2 mmol/L l‐glutamine, 100 U/mL penicillin, 100 µg/ml streptomycin, 1 mmol/L sodium pyruvate, and 10 mmol/L 4‐(2‐hydroxyethyl)‐1‐piperazineethanesulfonic acid [HEPES] buffer, unless otherwise stated. The mouse MM tumor cell line 5TGM1 (expressing a dual green fluorescent protein (GFP) and firefly luciferase reporter construct^31^, ^32^) was maintained in Iscove's modified Dulbecco's medium (IMDM) with 20% fetal calf serum (FCS; Thermo Fisher Scientific) and supplements. The human MM tumor cell line RPMI‐8226 was maintained in Roswell Park Memorial Institute 1640 (RPMI‐1640) with 10% FCS and supplements. The human bone marrow EC line TrHBMEC (kindly provided by Prof. Babette B. Weksler, Weill Cornell Medical College),^33^ hereafter called bone marrow EC (BMEC), was maintained in M199 medium with 20% FCS and supplements (BMEC medium), as previously described.^34^ All cell lines were maintained at 37°C in a humidified atmosphere with 5% CO_2_.

### 2.2 Drugs

For in vitro experiments, LCRF‐0006 (kindly provided by Crocus Laboratories) was solubilized in dimethyl sulfoxide (DMSO) (Merck). For in vivo experiments, LCRF‐0006 was solubilized in saline containing 40% 2‐hydroxypropyl‐β‐cyclodextrin (2‐HP‐β‐CD; Merck). Bortezomib (VELCADE^®^; Janssen‐Cilag Pty Ltd) was reconstituted at a final concentration of 0.028% DMSO for in vitro studies and 1.33% DMSO for in vivo studies.

### 2.3 Cell apoptosis assays

To assess the effect of LCRF‐0006 on 5TGM1 cell viability in vitro, 5TGM1 cells were cultured at 1 x 10^5^ cells/ml with LCRF‐0006, or vehicle alone, in 12‐well plates. After 3 days, 1 × 10^5^ cells/test were washed in IMDM with 20% FCS and additives, then spun and resuspended in 20 µL annexin V binding buffer (Hank's balanced salt solution with 1% HEPES and 5 mmol/L CaCl_2_) containing 0.075 µg/mL annexin V‐PE (BioLegend) and 10% (v/v) 7‐AAD (Beckman Coulter), and stained for 20 minutes at 4°C. Cells were then diluted with 200 µL ice‐cold annexin V binding buffer, immediately run on a LSRFortessa™ X‐20 flow cytometer (BD) and analyzed using FlowJo V.10.0.8 software (FlowJo, LLC). For single‐stained positive controls, 5TGM1 cells were treated with DMSO (early apoptosis control) or 80% ethanol (dead cell control) for 10 minutes. For drug synergy assays, 5TGM1 cells were cultured, as described above, in combinations of various concentrations of LCRF‐0006 and bortezomib. After 24 hours, 1 × 10^5^ cells from each condition were stained and analyzed, as described above. Drug synergy was defined as a coefficient of drug interaction (CDI) value of less than 0.7, where CDI was the actual viability of 5TGM1 cells treated with the drug combination (AB) divided by their predicted viability (calculated as the product of 5TGM1 cell viabilities treated with each drug alone [A*B]).

To assess the effect of LCRF‐0006 on BMEC viability in vitro, BMECs were seeded at 1.2 × 10^5^ cells/well (2.4 × 10^5^ cells/mL) in BMEC medium in 24‐well plates and cultured for 24 hours. Confluent BMEC monolayers were then cultured with LCRF‐0006 or vehicle in M199 medium with 15% FCS and supplements (as previously described^34^). After 24 hours, BMECs were trypsinized and 1 × 10^5^ cells/test were washed in M199 medium with 15% FCS and supplements, and prepared and analyzed for cell viability assessment, as described above.

### 2.4 EC monolayer retraction and recovery assay and N‐cadherin immunofluorescence staining

BMECs were seeded onto gelatinized 96‐well plates (5 × 10^4^ cells/well) and grown to confluence over 24 hours. Cells were then treated with LCRF‐0006 in IMDM with 2% FCS and additives for 1 hour and imaged using an Olympus CKX41 inverted microscope and DP21 imaging system. After gently washing twice in IMDM with 2% FCS and additives, cells were allowed to recover for 1 hour in M199 with 20% FCS and BMEC supplements, and were again imaged. BMEC monolayer confluency was assessed using cellSens Entry 1.11 software (Olympus). For N‐cadherin immunofluorescence staining at EC junctions, confluent BMEC cultures on 8‐well glass slide chamber slides (Merck) were treated with LCRF‐0006 for 1 hour, as described above. Cells were then fixed in 4% paraformaldehyde for 10 mins at room temperature (RT), rinsed in phosphate‐buffered saline (PBS) and permeabilized with 0.01% Triton‐X‐100 for 1 hour at RT. After blocking in 10% normal goat serum containing 1% bovine serum albumin (BSA) for 1 hour at RT, cells were incubated with mouse monoclonal anti‐N‐cadherin antibody (BD; 1:500) in blocking buffer overnight at 4°C. Cells were then rinsed in PBS and incubated with goat anti‐mouse IgG Alexa Fluor 647 secondary antibody (Thermo Fisher Scientific; 1:2000) and 1 µg/ml Hoechst 33342 (Thermo Fisher Scientific) in PBS for 1 hour at RT. Fluorescent images were acquired using an Olympus FV3000 confocal microscope.

### 2.5 EC monolayer permeability assay

Bone marrow ECs (5 × 10^4^ cells/well) were seeded onto gelatinized 0.4 µm 6.5 mm trans‐wells (Corning) and grown to confluence over 24 hours. BMECs were then treated with LCRF‐0006 in IMDM with 2% FCS and additives for 1 hour and gently washed twice in serum‐free IMDM with additives. To measure EC monolayer permeability, 1 mg/mL 70 kDa FITC‐dextran (Merck) in phenol red‐free IMDM with 2% FCS and additives was added to the trans‐wells and fluorescence in the bottom chamber (containing phenol red‐free IMDM with 2% FCS and additives) was assessed after 1 hour using a FLUOstar^®^ Omega microplate reader (BMG LABTECH).

### 2.6 EC tube disruption assays

Endothelial cell tubes were pre‐formed on growth factor‐reduced Matrigel^®^ matrix (Corning) in a 96‐well plate by seeding 3.5 × 10^4^ BMECs in a 50:50 mix of BMEC medium and conditioned media from the human MM cell line RPMI‐8226 as a stimulant, as described previously.^35^ To investigate the effect of LCRF‐0006 on EC tube integrity, immature (5‐hour‐old) or established (24‐hour‐old) tubes were then treated with up 200 µg/mL LCRF‐0006. Tubes were then imaged over a 24‐hour period using an Olympus CKX41 inverted microscope and DP21 imaging system, and analyzed using Olympus cellSens Entry 1.11 software.

### 2.7 Animal studies

All animal procedures were performed in accordance with guidelines approved by the South Australian Health and Medical Research Institute (SAHMRI) Animal Ethics Committee (SAM165). C57BL/KaLwRij mice (aged 6‐8 weeks) were used for all in vivo studies. For MM tumor studies, mice were inoculated with 5 × 10^5^ 5TGM1 cells in 100 µL PBS by intravenous injection (iv) via the tail vein. Total body tumor burden was assessed at days 14, 21 and 28 by bioluminescence imaging (BLI) using the IVIS^®^ Spectrum in vivo imaging system (Perkin Elmer), as previously described.^34^

All animals used for therapy studies were randomized by age, sex and, where appropriate, tumor burden. For monotherapy studies, mice were administered LCRF‐0006 (100 mg/kg/day) or vehicle alone by intraperitoneal injection (i.p.) (110‐150 µL volume), commencing at day 14 following establishment of disease until the conclusion of the experiment. For the drug combination studies, mice with established MM disease were administered LCRF‐0006 (100 mg/kg) or vehicle alone i.p. on day 14, followed by six cycles of LCRF‐0006 and bortezomib combination therapy (or relevant vehicle controls) over the remaining 14 days of the experiment. Each treatment cycle consisted of LCRF‐0006 (100 mg/kg) or 2‐HP‐β‐CD vehicle alone i.p. followed by low‐dose bortezomib (0.5 mg/kg)^36^ or 1.33% DMSO vehicle alone i.p. 1 hour later. At day 28, cardiac blood was collected into tubes containing 50 µL 0.5 mol/L ethylenediaminetetraacetic acid (EDTA) (pH 8.0) and complete blood counts were performed using a HEMAVET^®^950 automated blood analyser (Drew Scientific). Drug synergy was defined as a coefficient of drug interaction (CDI) value of less than 0.7, where CDI was the actual tumor burden in mice treated with the drug combination (AB) relative to mice treated with vehicles alone (as assessed by BLI), divided by the predicted tumor burden in mice (calculated as the product of tumor burden in mice treated with each drug alone [A*B]) relative to mice treated with vehicles alone (as assessed by BLI).

To assess the effect of LCRF‐0006 on the permeability of MM tumor‐associated vasculature to molecules with high affinity for plasma proteins, dynamic BLI was performed following administration of the firefly luciferase substrate D‐luciferin, which has high plasma protein binding affinity.^37^, ^38^, ^39^ Specifically, MM tumor‐bearing C57BL/KaLwRij mice at day 21 were administered LCRF‐0006 (100 mg/kg) or vehicle alone i.p. and, 1 hour later, mice were administered D‐luciferin (150 mg/kg) (Biosynth Carbosynth). Bioluminescence was then measured at 90‐second intervals over a 2‐hour time‐course. Total body bioluminescence signal (photon flux) over time was normalized to peak signal to normalize to tumor burden.

### 2.8 Cell composition analysis of compact bone

Compact bone was isolated from the long bones (ie, femora and tibiae) of both hind limbs from humanely killed mice and prepared for analysis of mesenchymal stromal cell (MSC), osteoprogenitor (OP), and osteoblast (OB) numbers, as previously described.^31^ Samples were run on a LSRFortessa™ X‐20 flow cytometer (BD) using FACSDiva™ software v8.0 (BD) and analyzed using FlowJo V.10.0.8 software (FlowJo, LLC; Ashland, OR). Non‐hematopoietic cell populations were defined as: Lin^−^CD45^−^CD31^+^ (ECs), Lin^−^CD45^−^CD31^−^Sca‐1^+^CD51^−^ (MSCs), Lin^−^CD45^−^CD31^−^Sca‐1^+^CD51^+^ (OPs) and Lin^−^CD45^−^CD31^−^Sca‐1^−^CD51^+^ (OBs).

### 2.9 Statistical analyses

For in vitro studies, statistical significance was calculated using a one‐way ANOVA with Dunnett's multiple comparisons test, or two‐way ANOVA with Sidak's multiple comparisons test. For in vivo studies, statistical significance was calculated using a Mann‐Whitney *U* test, Wilcoxon matched‐pairs signed rank test, Kruskal‐Wallis test with Dunn's multiple comparisons test, one‐way ANOVA with Holm‐Sidak's multiple comparisons test or two‐way ANOVA with Bonferroni's multiple comparisons test. Tumor burden data were log‐transformed prior to conducting statistical analyses. All statistical analyses were performed using GraphPad Prism^®^ v7.02 software (GraphPad Software, Inc).

## 3 RESULTS

### 3.1 LCRF‐0006 disrupts EC junctions and increases monolayer permeability at sub‐cytotoxic doses in vitro

**FIGURE 2:**
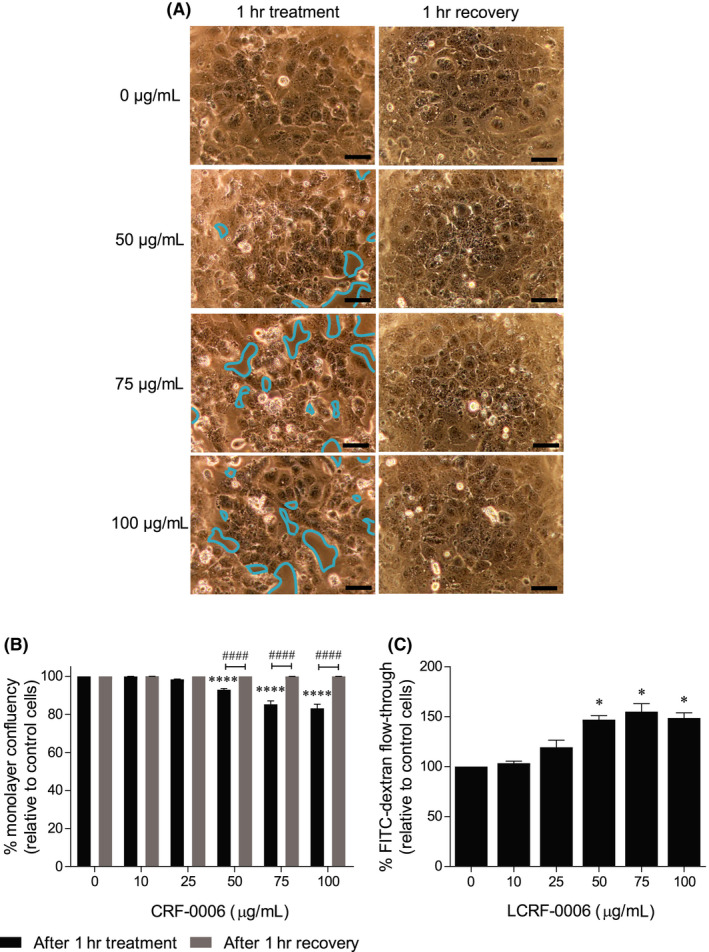
LCRF‐0006 disrupts BMEC monolayers and increases their permeability to 70 kDa FITC‐dextran in vitro. A, Confluent BMEC monolayers in 96‐well plates were treated with LCRF‐0006, or vehicle alone, for 1 h and then imaged. After removing LCRF‐0006, monolayers were gently washed, allowed to recover for 1 h and imaged again. Images shown are representative of three independent experiments. Scale bars depict 100 µm. B, BMEC monolayer confluency quantitated after treatment and recovery. Graph depicts mean ± SEM of three independent experiments. ****P < .0001 compared with monolayers treated with vehicle alone (0 µg/mL) ^####^ *P* < .0001 compared with monolayers after 1 h of recovery (two‐way ANOVA with Sidak's multiple comparisons test). C, Confluent BMEC monolayers established on 0.4 µm trans‐well membranes were treated with LCRF‐0006, or vehicle alone, for 1 h. Monolayers were then gently washed, 70 kDa FITC‐dextran (1 mg/mL) was added to the upper chamber of trans‐wells and FITC‐dextran flow‐through was assessed 1 h later by measuring fluorescence in the bottom chamber of triplicate trans‐wells. Graph depicts mean ± SEM of three independent experiments. *P < .05 compared with monolayers treated with vehicle alone (0 µg/mL) (one‐way ANOVA with Dunnett's multiple comparisons test). BMEC, bone marrow endothelial cell

Previous studies have shown that N‐cadherin antagonists rapidly compromise the integrity of confluent EC monolayers, resulting in increased vascular permeability in vitro.^21^, ^40^ In order to assess whether LCRF‐0006 disrupts EC monolayers, we treated confluent BMEC monolayers with LCRF‐0006 in vitro. Initially, we investigated whether LCRF‐0006 affected the viability of BMECs in vitro, using Annexin V (early apoptosis) and 7‐AAD (dead cell) staining. The treatment of confluent BMEC monolayers with LCRF‐0006 for 24 hours had no significant effect on BMEC viability in comparison to vehicle‐treated monolayers, as assessed by the AnnexinV^neg^ 7‐AAD^neg^ cell population, at concentrations up to at least 150 µg/mL (Figure S1). However, LCRF‐0006 induced a dose‐dependent dissociation of EC junctions leading to EC retraction and rounding, and decreased monolayer confluency after 1 hour of treatment at concentrations of 50 µg/mL and greater, when compared with vehicle treatment (*P* < .0001; Figure 2A,B). LCRF‐0006 also induced a dose‐dependent reduction in N‐cadherin staining intensity at EC junctions at concentrations of 50 µg/mL and greater (Figure S2). Notably, the effects of LCRF‐0006 were reversible, as monolayers treated with up to 100 µg/mL LCRF‐0006 completely reformed within 1 hour of drug removal (Figure 2A,B). Previous studies have shown that EC retraction, rounding and gap formation is associated with increased vascular permeability to molecules with high molecular mass in vitro.^41^, ^42^, ^43^, ^44^ To this end, we assessed the effect of LCRF‐0006‐mediated BMEC monolayer disruption on vascular permeability to high molecular mass molecules in a trans‐well flow‐through assay using 70 kDa FITC‐dextran. The pre‐treatment of confluent BMEC monolayers with LCRF‐0006 for 1 hour significantly increased BMEC monolayer permeability to 70 kDa FITC‐dextran in a dose‐dependent manner, when compared with vehicle‐treatment (*P* < .05) (Figure 2C).

### 3.2 LCRF‐0006 disrupts EC tube integrity in vitro

**FIGURE 3:**
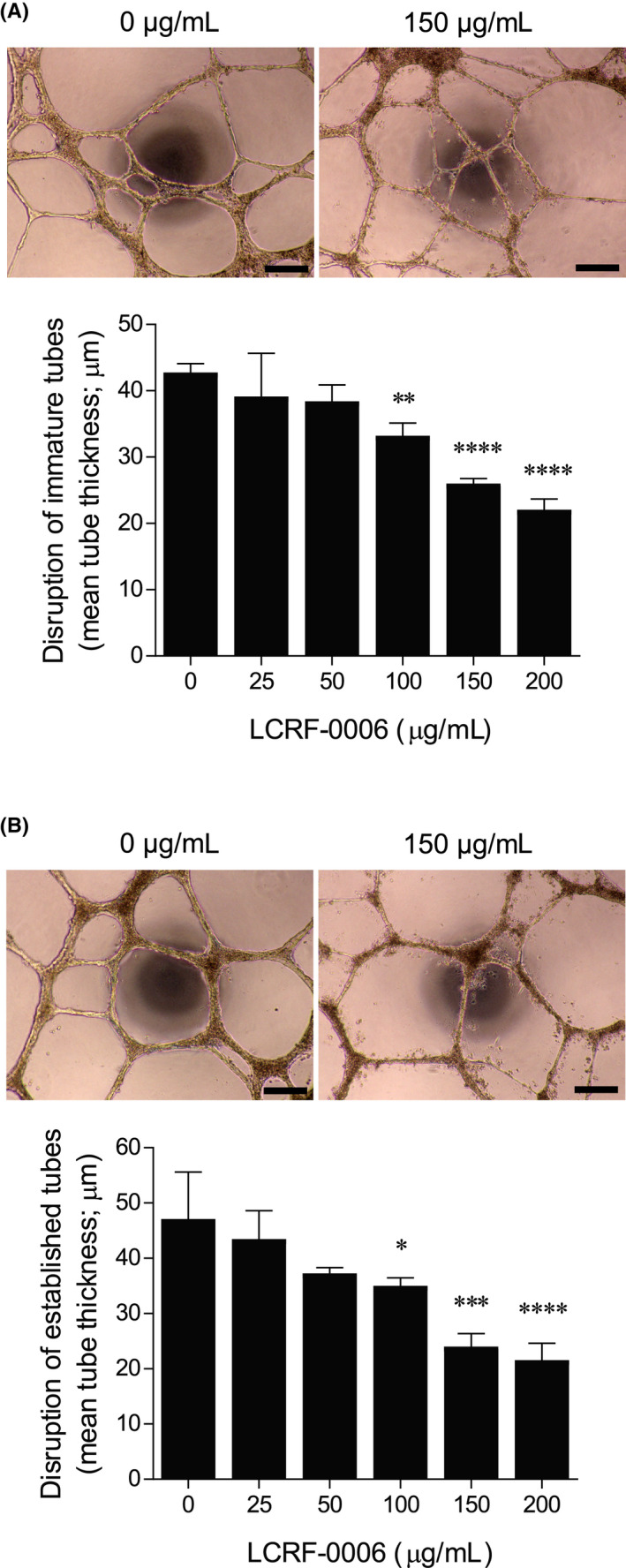
LCRF‐0006 disrupts immature and established endothelial tubes in vitro. BMECs were cultured on growth factor‐reduced Matrigel^®^ matrix in a 96‐well plate and endothelial tube formation was induced using a 50:50 mix of BMEC culture medium and RPMI‐8226 conditioned medium. A, Immature (5‐hour‐old) or (B) established (24‐hour‐old) tubes were treated with LCRF‐0006 for 24 h, imaged and mean tube thickness was quantitated. Images shown are representative of two independent experiments. Scale bars depict 250 µm. Graphs depict mean ± range of two independent experiments. *P < .05 **P < .01 ***P < .001 ****P < .0001 compared with tubes treated with vehicle alone (0 µg/mL) (one‐way ANOVA with Dunnett's multiple comparisons test). BMEC, bone marrow endothelial cell

We then assessed the effect of LCRF‐0006 on pre‐formed three‐dimensional EC tubes grown in a basement membrane‐like Matrigel^®^ matrix in vitro. Initially, we assessed the effect of LCRF‐0006 on immature (5‐hour‐old) EC tubes. Vehicle‐treated immature tubes matured normally into networks of thick tubes with smooth morphology over 24 hours (Figure S3A,B). In contrast, concentrations of 100 µg/mL LCRF‐0006 and greater compromised tube maturation, with EC rounding and detachment from tubes observed, resulting in a significant reduction in tube thickness after 24 hours (*P* < .0001) (Figure 3A and Figure S3A,B). However, addition of LCRF‐0006 to immature tubes did not reduce the total length of EC tubes after 24 hours (data not shown). Notably, the initial effects of LCRF‐0006 treatment were rapid, with BMEC retraction and rounding seen as early as 30 minutes following LCRF‐0006 addition (data not shown), consistent with EC gap formation.^41^, ^45^, ^46^ LCRF‐0006 was then added to established (24‐hour‐old) networks of EC tubes. Similar to the effects on immature tubes, treatment with 100 µg/mL LCRF‐0006 and greater for 24 hours resulted in a loss of tube smoothness and a significant reduction of tube thickness (*P* < .0001) (Figure 3B and Figure S4A,B), without affecting tube length (data not shown).

### 3.3 LCRF‐0006 is well tolerated in vivo and does not affect the cellular composition of the bone marrow microenvironment

Given the effects of LCRF‐0006 on EC monolayer integrity and vascular permeability in vitro, we then postulated that LCRF‐0006 may increase vascular permeability in tumors and enhance tumor response to anti‐cancer agents in vivo, in line with previous findings using ADH‐1.^21^ Previous studies have shown that 50 mg/kg LCRF‐0006 treatment by i.p. injection for 5 days in CD‐1 mice is well tolerated, with no signs of toxicity (plasma concentration peaks ~1 hour after i.p. injection; plasma concentration 1 hour after i.p. injection = 113.4 ± 25.47 µg/ml (mean ± SD); Adherex Technologies Inc (now known as Fennec Pharmaceuticals Inc) (personal communication)). Prior to conducting studies in C57BL/KaLwRij MM tumor‐bearing mice, we assessed the safety and toxicity of daily LCRF‐0006 treatment in tumor‐naive mice. C57BL/KaLwRij mice treated with 100 mg/kg/day LCRF‐0006 for 28 days tolerated the dosing regimen well, with no effects on weight (Figure S5A) or other adverse effects observed when compared with vehicle‐treated animals. In addition, complete blood counts were unaffected by 28 days of LCRF‐0006 treatment, in comparison with vehicle‐treated, or untreated, mice (Table S1) suggesting that hematopoiesis was unaffected. As N‐cadherin has been shown to impede OB differentiation,^47^, ^48^, ^49^, ^50^ we also assessed whether LCRF‐0006 affected the proportion of MSCs, OPs and OBs within the long bones of LCRF‐0006‐treated mice. Daily LCRF‐0006 treatment for 28 days had no significant effect on the proportion of MSCs, OPs and OBs within the Lin^−^CD45^−^CD31^−^ fraction of compact bone, when compared with vehicle‐treated animals (Figure S5B‐D). Moreover, LCRF‐0006 did not affect the proportion of CD31^+^ ECs within the Lin^−^CD45^−^ fraction of compact bone or bone marrow, relative to that of vehicle‐treated controls (Figure S5E,F). In line with these findings, histological analysis revealed LCRF‐0006 had no overt effects on blood vessel number or vascular morphology (data not shown).

### 3.4 LCRF‐0006 increases the permeability of MM tumor‐associated vasculature

To investigate whether LCRF‐0006 increases the permeability of tumor‐associated vasculature in vivo, we performed dynamic BLI using the firefly luciferase substrate D‐luciferin that displays high affinity for plasma proteins.^37^ Dynamic BLI enables the measurement of the relative uptake of D‐luciferin by luciferase‐expressing tumor cells, over time, with the bioluminescence signal reflecting the extravasation of D‐luciferin from the vasculature to the tumor.^38^, ^39^

**FIGURE 4:**
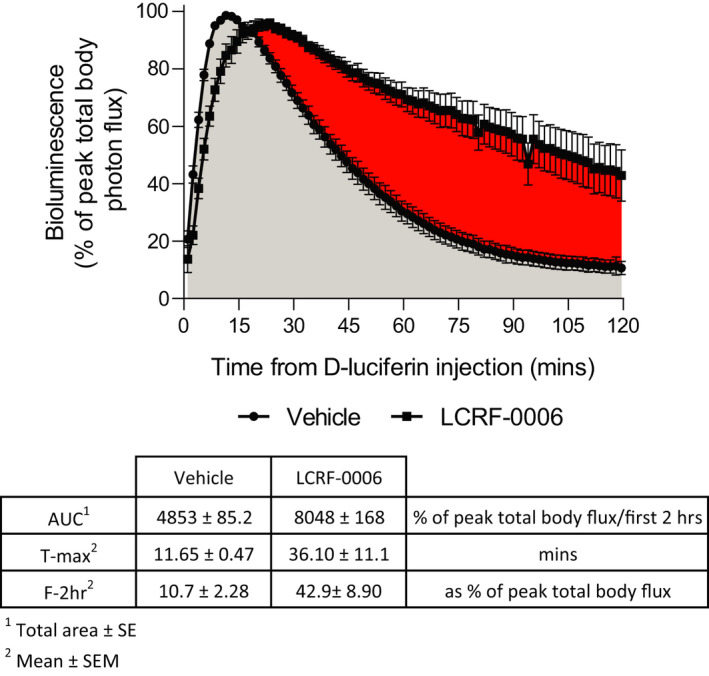
LCRF‐0006 increases the permeability of MM tumor‐associated vasculature. Tumor‐bearing C57BL/KaLwRij mice (week 3 tumor) were injected i.p. with 100 mg/kg LCRF‐0006 or 2‐HP‐b‐CD vehicle alone followed by D‐luciferin 1 h later. Bioluminescence was then measured at 90‐second intervals over a 2‐hour time‐course to monitor uptake of D‐luciferin into the tumor. Curves represent the mean ± SEM of bioluminescence over time as a percentage of peak total body photon flux reached over a 2‐hour scan (n = 10 mice/treatment). Shaded areas show bioluminescence AUC following treatment with 2‐HP‐b‐CD vehicle (gray) in comparison to treatment with LCRF‐0006 (red). Inset shows quantified area under the curve (AUC), time of peak total body flux (T‐max) and total body flux 2 h after D‐luciferin injection (F‐2 h). MM, multiple myeloma

The plasma concentration of LCRF‐0006 in mice peaks approximately 1 hour after i.p. injection (Fennec Pharmaceuticals Inc (personal communication)). In line with this, we injected MM tumor‐bearing C57BL/KaLwRij mice (21 days after 5TGM1 cell injection) with D‐luciferin 1 hour after treatment with LCRF‐0006 or 2‐HP‐β‐CD vehicle alone. When mice were treated with vehicle alone, total body bioluminescence signal (photon flux) peaked at 11.7 ± 0.47 minutes (mean ± SEM) after D‐luciferin injection. In contrast, when mice were treated with 100 mg/kg LCRF‐0006, total body photon flux peaked after 36.1 ± 11.1 minutes (mean ± SEM) (*P* < .01; Wilcoxon matched‐pairs signed rank test). However, LCRF‐0006 treatment resulted in a more sustained bioluminescence signal over time, with total body photon flux at 2 hours being significantly higher in mice with LCRF‐0006 treatment, in comparison to vehicle treatment (42.9 ± 8.90% vs 10.7 ± 2.28% [mean ± SEM]) (*P* < .01; Wilcoxon matched‐pairs signed rank test). Moreover, the cumulative bioluminescence signal (normalized to peak signal) over 2 hours following D‐luciferin injection was markedly higher when mice were treated with LCRF‐0006, in comparison to vehicle treatment (area under curve (AUC): 8048 ± 168.3 (total area ± SE) [95% CI: 7718‐8378]) vs 4853 ± 85.20 (total area ± SE) [95% CI: 4686‐5020]) (Figure 4). This suggests that LCRF‐0006 significantly increases D‐luciferin extravasation at MM tumor sites.

### 3.5 LCRF‐0006 and bortezomib combination therapy synergistically induces MM tumor regression in mice with established disease

**FIGURE 5:**
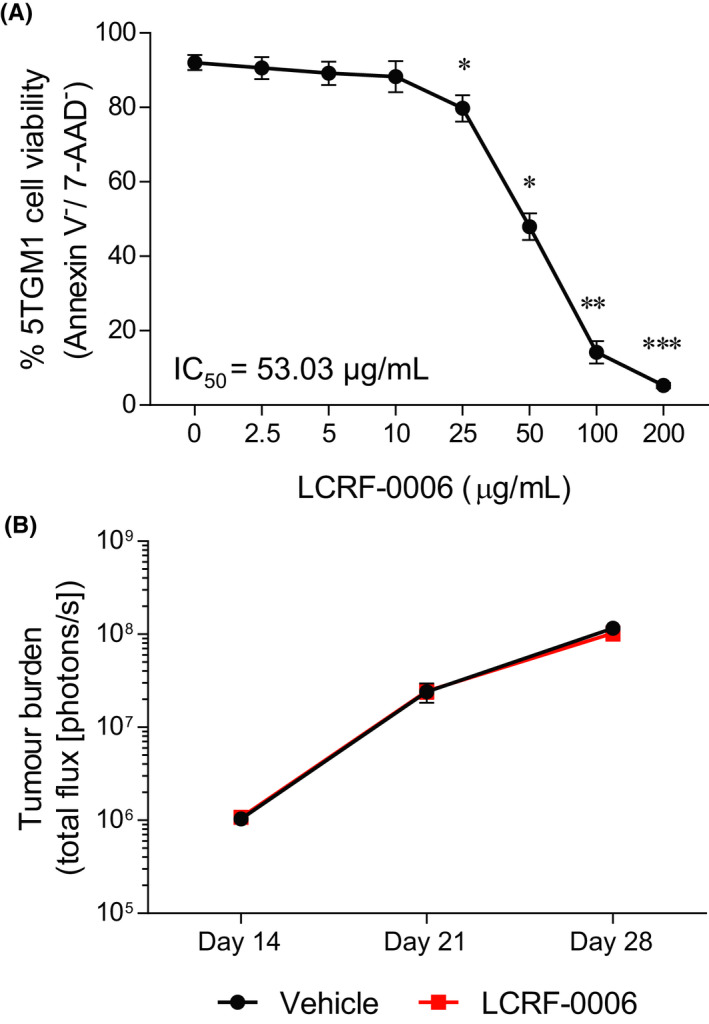
LCRF‐0006 monotherapy induces 5TGM1 cell apoptosis in vitro but does not inhibit tumor progression in vivo. A, The viability of 5TGM1 cells following in vitro culture in the presence of LCRF‐0006 for 72 h was assessed by flow cytometry following annexin V and 7‐AAD staining. Graph depicts mean ± SEM of three independent experiments. *P < .05 **P < .01 ***P < .001 compared with 5TGM1 cells treated with vehicle alone (0 µg/mL) (one‐way ANOVA with Dunnett's multiple comparisons test). B, C57BL/KaLwRij mice with established MM (day 14 post‐5TGM1 cell injection) were treated with LCRF‐0006 (100 mg/kg/day) or vehicle (CD) alone i.p. for 14 d. Tumor burden was assessed at day 14, 21 and 28 by BLI. Graph depicts mean ± SEM. n = 12 mice/treatment group. Data are not statistically significant (two‐way ANOVA). BLI, bioluminescence imaging. MM, multiple myeloma

Previous studies have reported the utility of ADH‐1 in the inhibition of tumor establishment and growth in a range of pre‐clinical mouse models including pancreatic cancer, lung cancer and MM.^34^, ^51^, ^52^ In addition to increasing the permeability of tumor‐associated vasculature,^21^, ^52^ ADH‐1 has been shown to induce apoptosis in several N‐cadherin‐expressing cancer cell types in vitro,^51^, ^53^, ^54^ suggesting that it may suppress tumor progression through direct effects on tumor cells. In order to investigate the anti‐cancer properties of LCRF‐0006, we initially assessed the effect of LCRF‐0006 treatment on the viability of the N‐cadherin‐expressing mouse MM cell line 5TGM1 in vitro. LCRF‐0006 treatment significantly induced 5TGM1 cell apoptosis at concentrations of 25 µg/mL and greater after 72 hours (*P* < .01; IC_50_ = 53.03 µg/mL) (Figure 5A). However, daily treatment of 5TGM1 tumor‐bearing C57BL/KaLwRij mice with 100 mg/kg LCRF‐0006 had no effect on tumor burden by day 28, as assessed by BLI , compared with vehicle‐treated controls (Figure 5B). Similar to MM tumor‐naive mice, LCRF‐0006 treatment also had no effect on the proportion of ECs within compact bone or bone marrow (Figure S6A,B), or the proportion of MSCs, OPs and OBs within compact bone (data not shown), in tumor‐bearing mice, when compared with vehicle‐treated animals.

**FIGURE 6:**
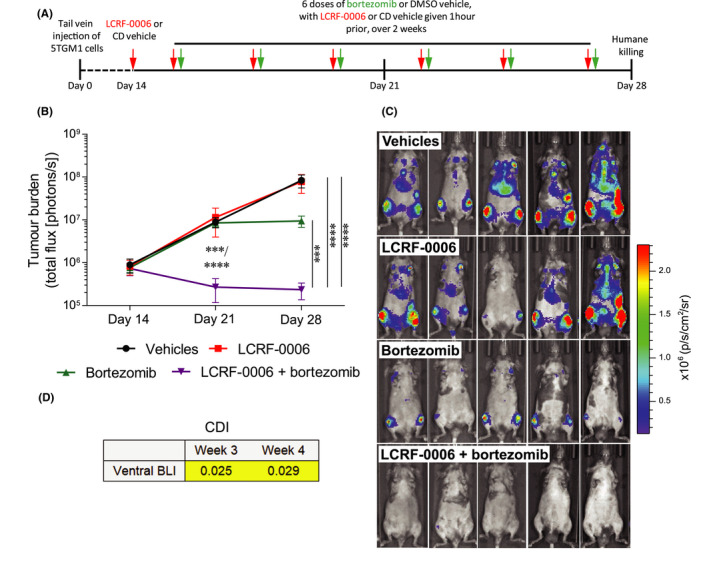
The combination of LCRF‐0006 and bortezomib synergistically increases MM tumor response in vivo. A, Schematic representation of treatment regimen. C57BL/KaLwRij mice with established MM (day 14 post‐5TGM1 cell injection) were treated with LCRF‐0006 (100 mg/kg) and bortezomib (0.5 mg/kg) (LCRF‐0006 + bortezomib), LCRF‐0006 and DMSO vehicle (LCRF‐0006), 2‐HP‐b‐CD vehicle and bortezomib (bortezomib) or 2‐HP‐b‐CD and DMSO vehicles (vehicles) i.p., as per treatment regimen. B, Tumor burden was assessed at day 14, 21 and 28 by BLI. Graph depicts mean ± SEM. n = 5‐6 mice/treatment group. ***P < .001 ****P < .0001 compared with mice treated with LCRF‐0006, bortezomib or vehicles (two‐way ANOVA with Tukey's multiple comparisons test). C, BLI images of mice treated with vehicles, LCRF‐0006, bortezomib or LCRF‐0006 + bortezomib, at day 28. D, The coefficient of drug interaction (CDI) was calculated using BLI data at day 21 and 28 to assess drug synergy. CDI <0.7 (shown in yellow boxes) indicates drug synergy. BLI, bioluminescence imaging. MM, multiple myeloma

The ability of ADH‐1 to augment vascular permeability resulted in enhanced tumor delivery and anti‐cancer effectiveness of the chemotherapeutic drug melphalan, which has a high affinity for plasma proteins.^21^ In light of the vascular permeability‐enhancing effects we observed with LCRF‐0006 in vitro and in vivo, we speculated that LCRF‐0006 may similarly enhance the delivery of anti‐cancer drugs with high plasma protein binding affinity to tumor sites in vivo, thereby increasing the effectiveness of the drug. To this end, MM tumor‐bearing C57BL/KaLwRij mice were administered three cycles of combination therapy per week for 2 weeks, whereby one cycle consisted of LCRF‐0006 (100 mg/kg) followed by a low dose of the anti‐MM agent bortezomib (0.5 mg/kg) 1 hour later (Figure 6A). Low‐dose bortezomib or LCRF‐0006 alone had no effect on tumor burden by day 28, in comparison to mice treated with vehicles alone. Importantly, mice which received the combination of LCRF‐0006 and low‐dose bortezomib had significantly lower tumor burden at days 21 and 28 compared with mice treated with bortezomib alone, LCRF‐0006 alone or vehicles only (all *P* < .001) (Figure 6B,C). Moreover, the combination therapy resulted in regression of tumor burden in 100% of treated mice at days 21 and 28, compared with tumor levels prior to therapy, with 40% having no BLI‐detectable tumor by day 28. The CDI revealed that the anti‐MM effects of LCRF‐0006 and low‐dose bortezomib were synergistic (at days 21 and 28) (Figure 6D). Notably, the combination therapy regimen was well tolerated, and no adverse effects on weight or complete blood counts were observed in comparison to vehicle‐treated mice (data not shown). Similar to LCRF‐0006 alone, low‐dose bortezomib and the combination of LCRF‐0006 and low‐dose bortezomib had no effect on the proportion of MSCs, OPs and OBs within compact bone, when compared with vehicle‐treated animals (data not shown). In addition, the combination of LCRF‐0006 and low‐dose bortezomib did not affect the proportion of CD31^+^ ECs within compact bone or bone marrow in mice with established disease, relative to vehicle‐treated control mice (data not shown).

### 3.6 LCRF‐0006 and bortezomib synergistically induce 5TGM1 cell apoptosis in vitro

**FIGURE 7:**
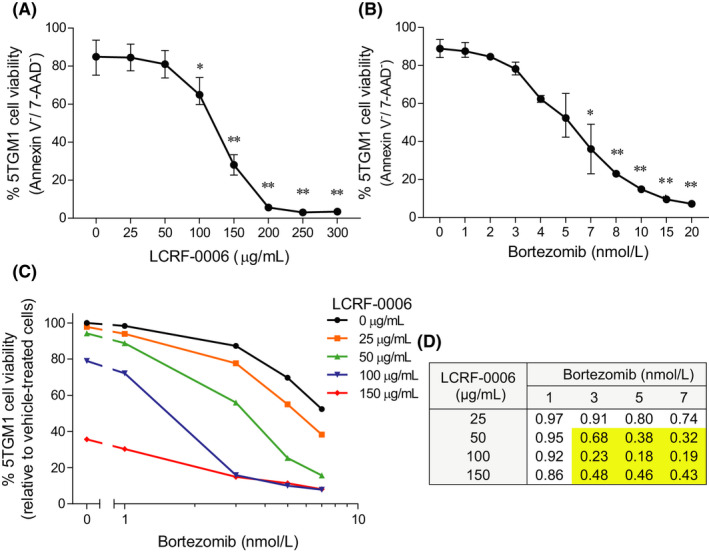
LCRF‐0006 synergistically increases bortezomib‐induced 5TGM1 cell apoptosis in vitro. The viability of 5TGM1 cells following in vitro culture in the presence of increasing concentrations of (A) LCRF‐0006 or (B) bortezomib for 24 h was assessed by flow cytometry following annexin V and 7‐AAD staining. Graphs depict mean ± SEM of three independent experiments. *P < .05 **P < .01   compared with 5TGM1 cells treated with vehicle alone (0 µg/mL) (one‐way ANOVA with Dunnett's multiple comparisons test). C, Graphical representation of 5TGM1 cell viability following in vitro culture in the presence of bortezomib and increasing concentrations of LCRF‐0006 for 24 h (as assessed by flow cytometry following Annexin V and 7‐AAD staining), relative to 5TGM1 cells treated with vehicle alone. Data are representative of three independent experiments. D, The coefficient of drug interaction (CDI) was calculated using the relative viability of 5TGM1 cells after 24 h to assess drug synergy. CDI <0.7 (shown in yellow boxes) indicates drug synergy. Data are representative of three independent experiments

While we speculate that the synergistic effects observed in vivo may, in part, be mediated by LCRF‐0006‐enhanced tumor delivery of bortezomib, previous studies have also demonstrated the ability of ADH‐1 to enhance tumor response to chemotherapeutic agents independently of increasing drug delivery to tumors.^21^ To this end, we investigated the effect of LCRF‐0006 in combination with bortezomib on induction of 5TGM1 cell apoptosis in vitro*,* as assessed by Annexin V and 7‐AAD staining. Initially, we determined the doses of LCRF‐0006 (25‐150 µg/mL) and bortezomib (1‐7 nmol/L) which individually had minimal to moderate pro‐apoptotic effects on 5TGM1 cells, after 24 hours (Figure 7A,B). Notably, we found that LCRF‐0006 and bortezomib had synergistic effects on 5TGM1 cell apoptosis, with LCRF‐0006 concentrations of 50 µg/mL and above enhancing the sensitivity of 5TGM1 cells to bortezomib, after 24 hours (CDI <0.7) (Figure 7C,D).

## 4 DISCUSSION

In many cancers, including the bone marrow cancer MM, achieving an optimal tumor response to drug therapy is key to maximising a patient's long‐term progression‐free and overall survival.^55^ However, the ability to achieve optimal tumor dosing with anti‐cancer drugs is limited by transvascular access of the drugs to the site of tumor.^56^ Bortezomib is a standard‐of‐care, frontline therapy in MM patients, and is also commonly used as maintenance therapy and in the disease relapse setting.^57^, ^58^ Following clinical administration, over 80% of bortezomib is bound by plasma proteins including albumin (~70 kDa; 4‐15 nm in size),^59^, ^60^ likely restricting its transvascular flow in tissues with tight endothelial transport control. In support of this, rodent studies using radiolabeled bortezomib have shown that bortezomib is rapidly accumulated in tissues, such as kidney and liver, where fenestrated capillaries allow passage of molecules up to 15 and 180 nm in size, respectively.^61^, ^62^ In contrast, the bone marrow displays reduced and less rapid bortezomib accumulation,^62^, ^63^ where transvascular access of molecules over 5 nm in size is restricted by inter‐EC junctions.^61^ The association between bortezomib distribution and the inherent vascular permeability of tissues suggests that artificially increasing vascular permeability may facilitate an increase in the transvascular flow of bortezomib. Notably, structural abnormalities render tumor‐associated vasculature more sensitive to vascular‐modulating agents than normal vasculature.^19^, ^20^ Thus, vascular‐modulating agents may allow a selective increase in transvascular bortezemib flow within MM tumors, leading to increased drug accumulation at MM sites and improved drug effectiveness. In support of this, a nitric oxide delivery system has been shown to increase vascular permeability in tumor tissue, resulting in a selective increase in the delivery, and enhanced effectiveness, of nanoparticle albumin‐bound paclitaxel (Abraxane^®^) in pre‐clinical models of solid cancer with inherently high or low leakiness.^64^, ^65^ In contrast, nitric oxide inhibition has been shown to decrease tumor vascular permeability to albumin‐bound Evans blue dye.^66^, ^67^ Notably, previous studies have demonstrated the ability of the N‐cadherin antagonist peptide ADH‐1 to increase EC monolayer permeability in vitro, and rapidly enhance tumor blood vessel permeability to albumin‐bound Evans blue dye in pre‐clinical models of melanoma. Moreover, ADH‐1 improved melanoma tumor uptake of, and response to, the plasma protein‐binding chemotherapeutic agent melphalan.^21^, ^68^ Thus, ADH‐1, and ADH‐1‐like agents, may increase the delivery of other plasma protein‐bound anti‐cancer drugs to sites of tumor by increasing the permeability of tumor‐associated vasculature, thereby improving tumor response.

The objective of this study was to evaluate the vascular permeability‐enhancing and anti‐cancer properties of the small molecule ADH‐1 mimetic LCRF‐0006. In line with pre‐clinical studies in melanoma using ADH‐1, our dynamic BLI studies using D‐luciferin in the C57BL/KaLwRij/5TGM1 MM mouse model suggests that LCRF‐0006 similarly increases the permeability of tumor‐associated vasculature to molecules with high binding affinity for plasma proteins. D‐luciferin, like bortezomib, is a low molecular mass molecule (under 1 kDa) that, upon administration, is bound by plasma proteins^37^, ^59^, ^60^ leading to restricted transvascular flow in the bone marrow due to the 5nm upper pore limit of EC junctions within the tissue. The in vitro observation that LCRF‐0006 induced EC retraction and rounding, but that disrupted monolayers recovered following removal of LCRF‐0006, suggests that the effects of LCRF‐0006 on EC junctions are transient and reversible, in line with the proposed role of N‐cadherin in endothelial barrier closure.^69^, ^70^ Notably, our in vitro BMEC permeability studies using 70 kDa FITC‐dextran suggest that the disruption of EC junctions by LCRF‐0006 treatment enhances the transendothelial movement of molecules with a comparable size to albumin, supporting the idea that LCRF‐0006 increases the ability of albumin‐bound molecules to move out of the vasculature and into the surrounding tissues. Moreover, our in vitro studies suggest that the disruptive effects of LCRF‐0006 are not a consequence of EC apoptosis, which may eventuate in vessel collapse. This is consistent with our findings that LCRF‐0006 did not affect the EC composition of long bones or the overall vascular architecture of the bone marrow, either in the normal or tumor setting. Thus, the effects of LCRF‐0006 differ to the vascular‐disrupting effects of microtubule‐depolymerizing agents that induce necrosis of tumor‐associated vasculature.^19^, ^46^, ^71^ The ability of LCRF‐0006 to increase tumor delivery of plasma protein‐bound molecules is also distinct from anti‐angiogenic agents that aim to enhance drug access to tumors by normalizing tumor‐associated vasculature and increasing blood flow within the tumor.^19^ Interestingly, our dynamic BLI studies demonstrated that LCRF‐0006 increased cumulative bioluminescence in MM tumor‐bearing mice over a two‐hour period despite a delay in reaching peak bioluminescence signal. Blood flow is a critical determinant in the rate at which D‐luciferin is delivered to sites of tumor.^72^ Thus, we speculate that LCRF‐0006‐mediated disruption of EC junctions may slow tumor blood flow and the rate at which plasma protein‐bound molecules are delivered to tumor sites, while enhancing the cumulative delivery and transvascular flow of these molecules within tumors over time. Importantly, we observed that the initial effects of LCRF‐0006 on EC retraction and rounding were more rapid in immature EC tubes than established tubes in our in vitro assays. As tumor‐associated vasculature is relatively immature and structurally abnormal,^19^, ^20^ these findings suggest that tumor‐associated vasculature may have increased sensitivity to LCRF‐0006‐mediated disruption, compared with normal blood vessels.

Given LCRF‐0006 enhanced the cumulative delivery of D‐luciferin to MM tumors, and that ADH‐1 increased melanoma tumor delivery of, and response to, the plasma protein‐binding chemotherapeutic agent melphalan,^21^, ^68^ we hypothesized that LCRF‐0006 may similarly enhance MM tumor delivery of, and response to, bortezomib in vivo. Despite LCRF‐0006 lacking single‐agent anti‐tumor efficacy in the C57BL/KaLwRij/5TGM1 MM model, a synergistic tumor response was observed using the combination of LCRF‐0006 and a sub‐therapeutic dose of bortezomib in mice with established disease. In addition to LCRF‐0006 increasing vascular permeability in MM tumors, our in vitro data suggest that direct synergistic effects of LCRF‐0006 and bortezomib on 5TGM1 cell apoptosis are also likely to contribute to the strong synergism observed in vivo. While the mechanism(s) of synergy are unclear, N‐cadherin has been shown to increase pro‐survival signaling in cancer cells, including activation of Bcl‐2^73^, ^74^, ^75^ and NF‐кB^76^ pathways, known to play key roles in MM tumor cell survival and resistance to anti‐MM agents.^77^, ^78^, ^79^, ^80^, ^81^, ^82^ Thus, the potential inhibitory effects of LCRF‐0006 on Bcl‐2 and NF‐кB signaling in MM tumor cells warrants investigation in the future.

The bone marrow microenvironment is a complex milieu of stromal cells, extracellular matrix proteins, cytokines and growth factors that promotes MM cell growth, proliferation and survival. It is also well established that adhesive interactions of MM tumor cells with surrounding microenvironmental cells and extracellular matrix proteins (eg, via integrins) confers resistance to standard‐of‐care MM agents, including bortezomib.^83^, ^84^, ^85^, ^86^ While our analyses revealed that LCRF‐0006 treatment did not affect the relative proportion of MSCs, OPs and OBs within long bones, N‐cadherin is expressed by numerous cell types within the bone marrow milieu, including MSCs and OBs, and mediates MM plasma cell adhesion to OBs.^47^, ^48^, ^87^ As N‐cadherin antagonists have been shown to overcome bone marrow stromal cell‐mediated resistance to chemotherapeutic agents in chronic myeloid leukemia cells,^88^, ^89^ we therefore cannot exclude the possibility that LCRF‐0006 treatment may disrupt MM tumor cell interactions with the supportive bone marrow niche in vivo, thereby increasing tumor cell sensitivity to bortezomib.

The protective effects of the bone marrow microenvironment may also account for the lack of tumor response to LCRF‐0006 monotherapy in vivo, given the observed pro‐apoptotic effects of LCRF‐0006 on 5TGM1 cells in vitro as a monoculture. However, it is also plausible that the concentration of LCRF‐0006 within the tumor interstitium of mice after administration of drug at 100 mg/kg may not reach the concentration required for LCRF‐0006 induction of 5TGM1 cell apoptosis in vitro (IC_50_ = ~53 µg/mL). Whether a higher dose of LCRF‐0006 would have direct anti‐tumor effects on MM tumors in vivo requires further investigation.

Despite significant progress in the development of novel therapeutic agents and effective combination strategies over the past decade, MM is still largely considered to be incurable with most patients relapsing and ultimately succumbing to the disease.^58^ Notably, there is evidence, both in vitro and in pre‐clinical animal models, that the use of ADH‐1 represents a strategy to increase the effectiveness of existing anti‐cancer agents.^21^, ^68^, ^88^, ^89^ However, these anti‐cancer effects have not been fully replicated in Phase I/II clinical trials.^23^, ^24^ Given small molecule mimetics may offer increased therapeutic efficacy over their peptide counterparts,^25^, ^26^ the use of an ADH‐1 mimetic such as LCRF‐0006 is a rational proposition to enhance tumor delivery of plasma protein‐binding drugs and increase clinical effectiveness. Importantly, our study is the first to demonstrate the potential clinical utility of an ADH‐1 mimetic in the oncology setting. Our findings suggest that LCRF‐0006 may have utility in a combinatorial approach to significantly increase bortezomib effectiveness and enhance the depth of tumor response in MM patients. Future studies will address whether the combination therapy could be used to reduce the effective dose of bortezomib, in order to reduce the incidence and severity of its debilitating side‐effects, including peripheral neuropathy, while maintaining an optimal MM tumor response.

## AUTHOR CONTRIBUTIONS

KMM, CMC, DRH, JEN, and KSO performed the research; A.A and DLR provided technical expertise. KMM, K.V and ACWZ conceptualized and designed the research. OWB contributed to the experimental design. KMM, K.V and ACWZ analyzed the data and wrote the paper. All authors revised the manuscript.

## Supporting information

Fig S1

Fig S2

Fig S3

Fig S4

Fig S5

Fig S6

Table S1
