# Supplementary figures and images for: LCRF‐0006, a small molecule mimetic of the N‐cadherin antagonist peptide ADH‐1, synergistically increases multiple myeloma response to bortezomib
Source: FASEB Bioadv. 2020 Jun 15;2(6):339–53. doi: 10.1096/fba.2019-00073 (PMC7325588; doi:10.1096/fba.2019-00073)

Supp. Fig. 1

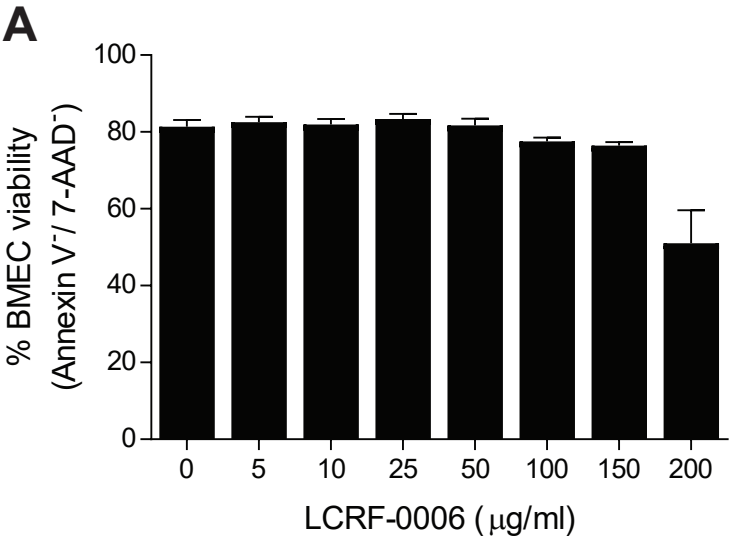

Supplement: Supplementary file 1 — Fig S1 [file FBA2-2-339-s001.pdf]

**Supp. Fig. 2**

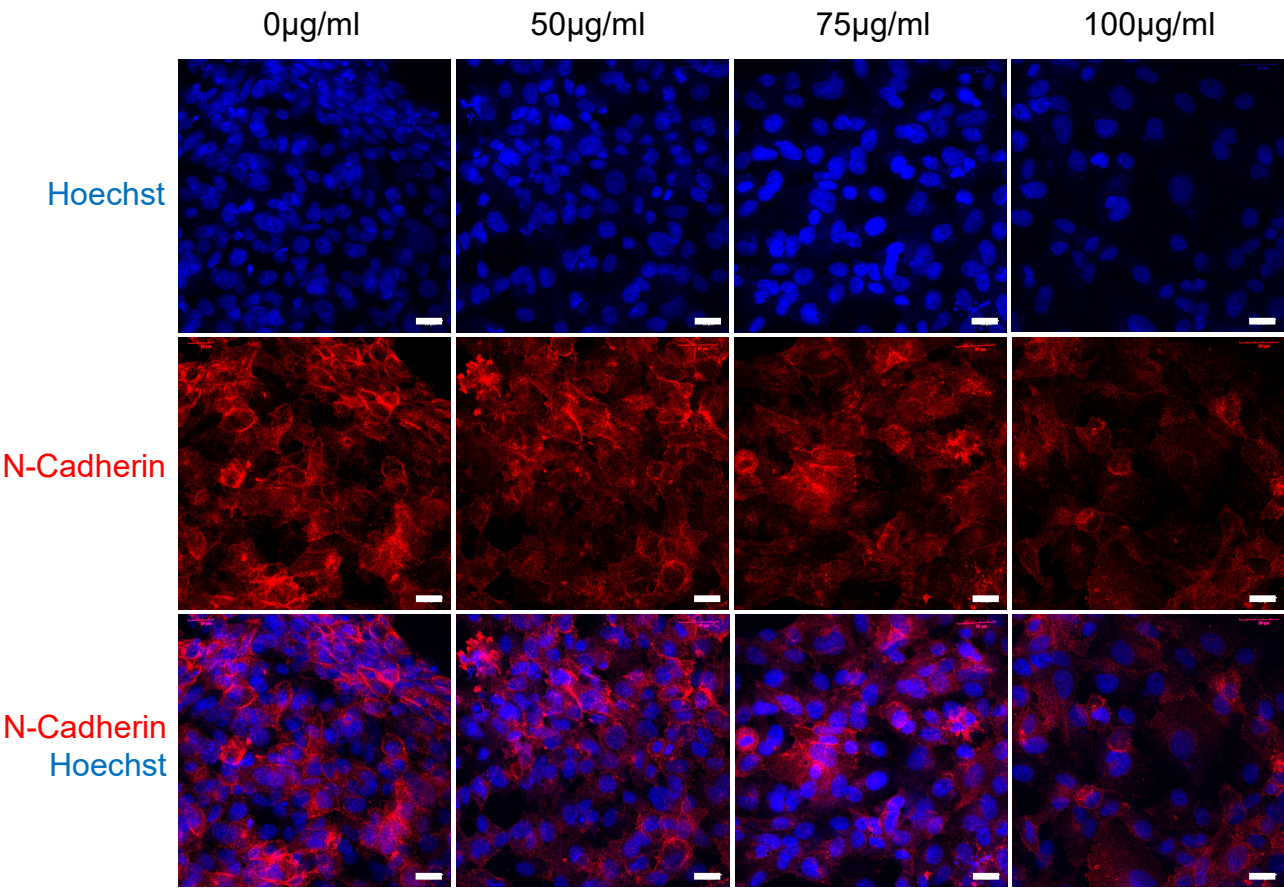

Supplement: Supplementary file 2 — Fig S2 [file FBA2-2-339-s002.pdf]

## Supp. Fig. 3

**A**

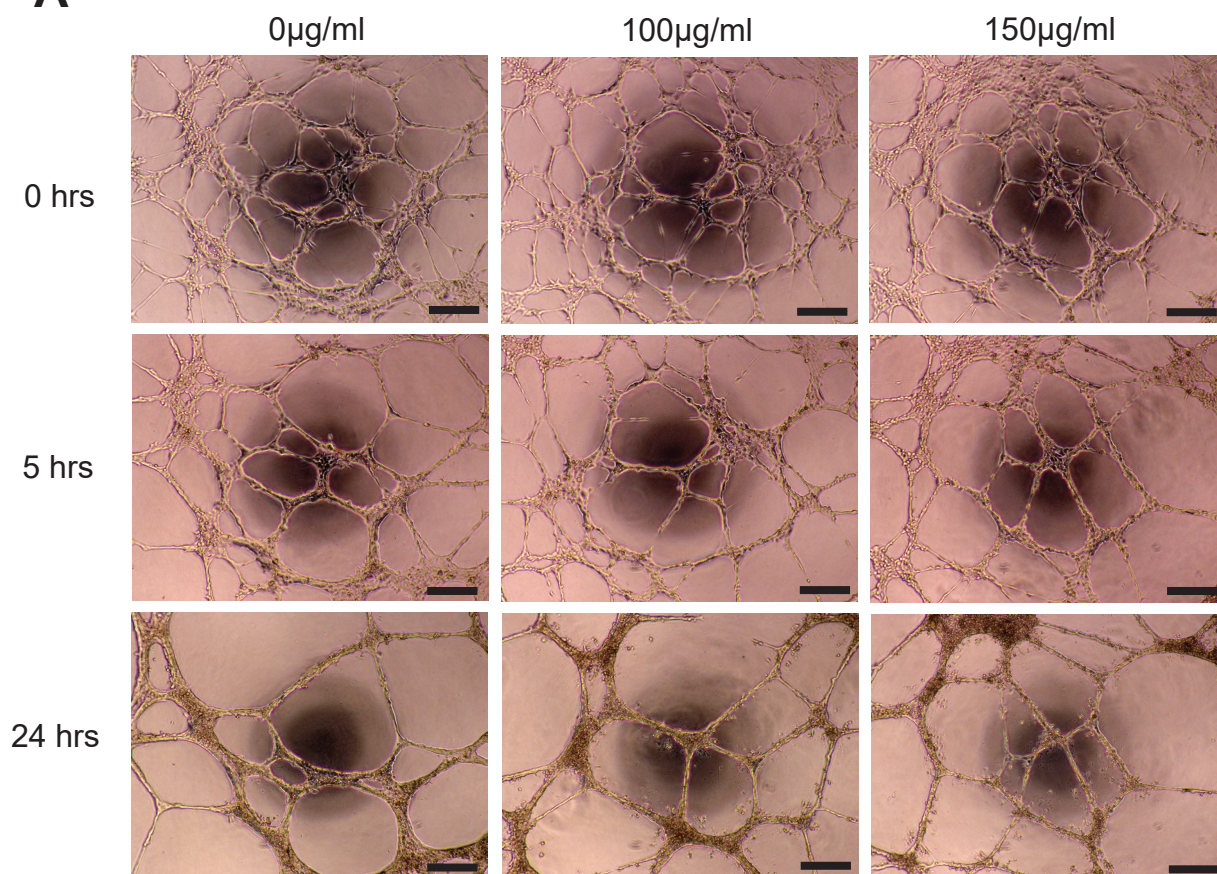

**B**

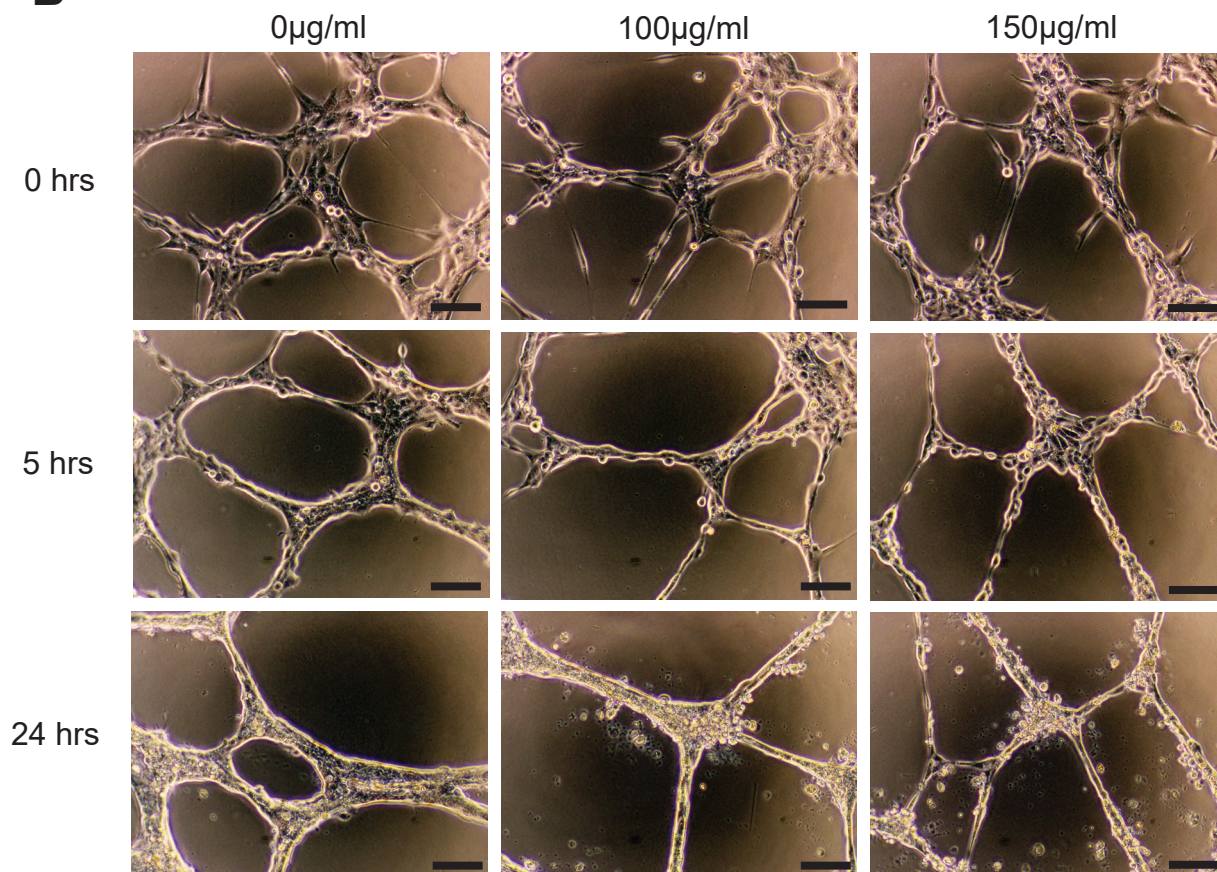

Supplement: Supplementary file 3 — Fig S3 [file FBA2-2-339-s003.pdf]

Supp. Fig. 4

**A**

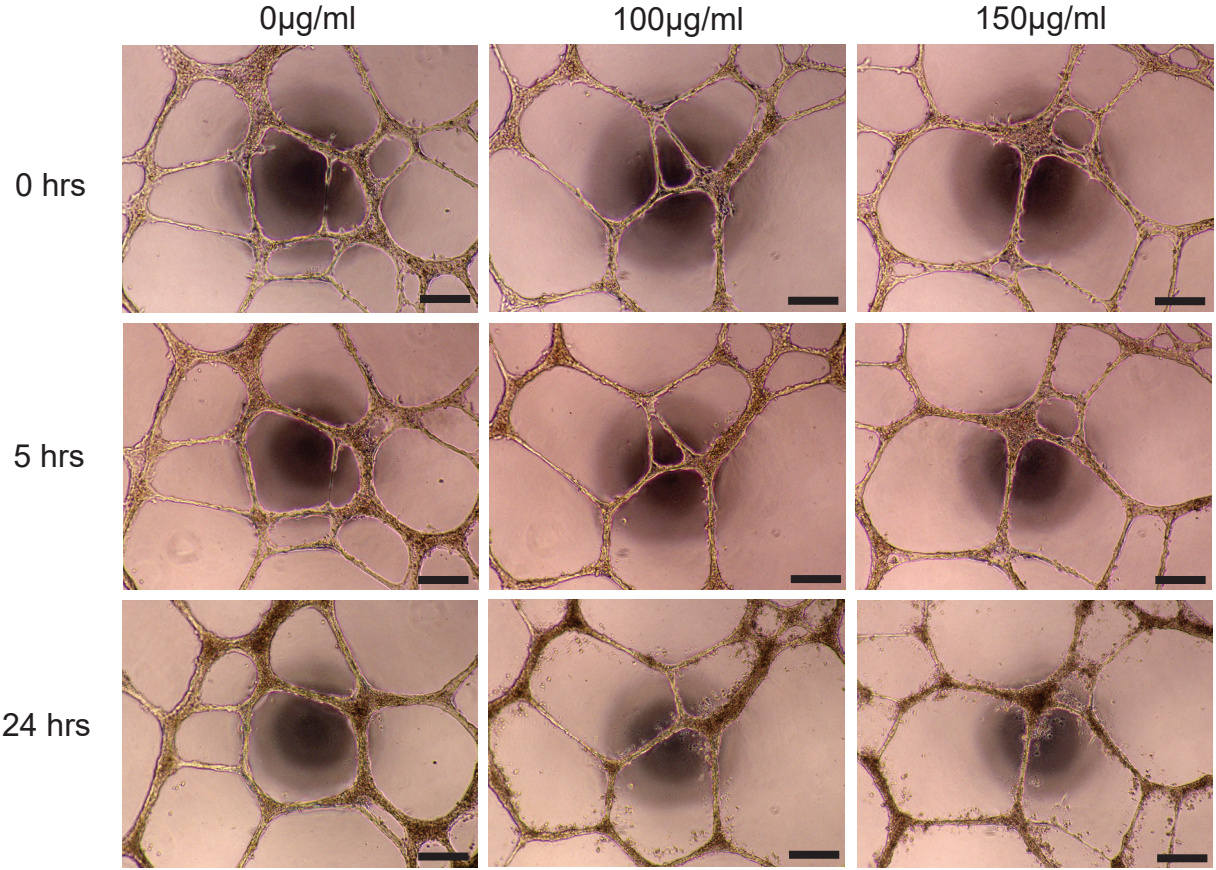

**B**

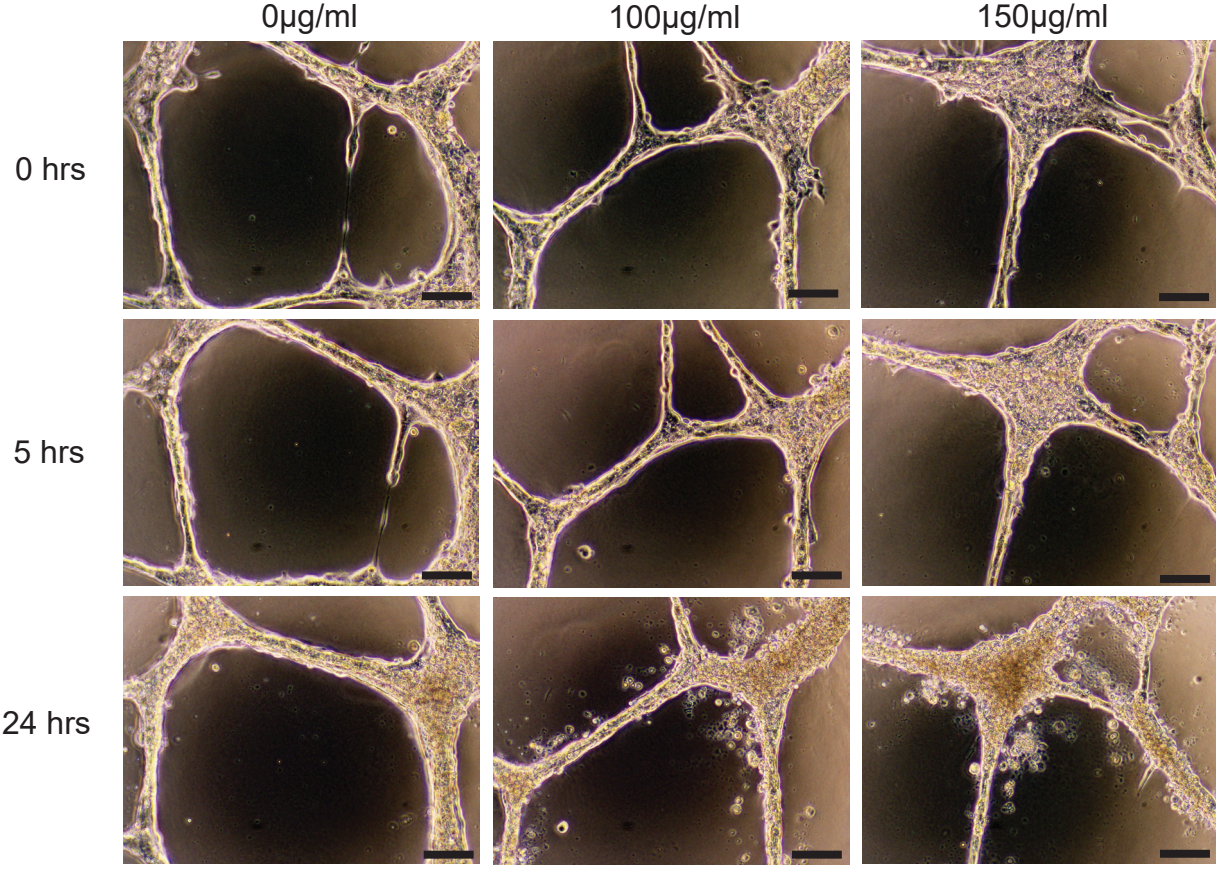

Supplement: Supplementary file 4 — Fig S4 [file FBA2-2-339-s004.pdf]

**Supp. Fig. 5**

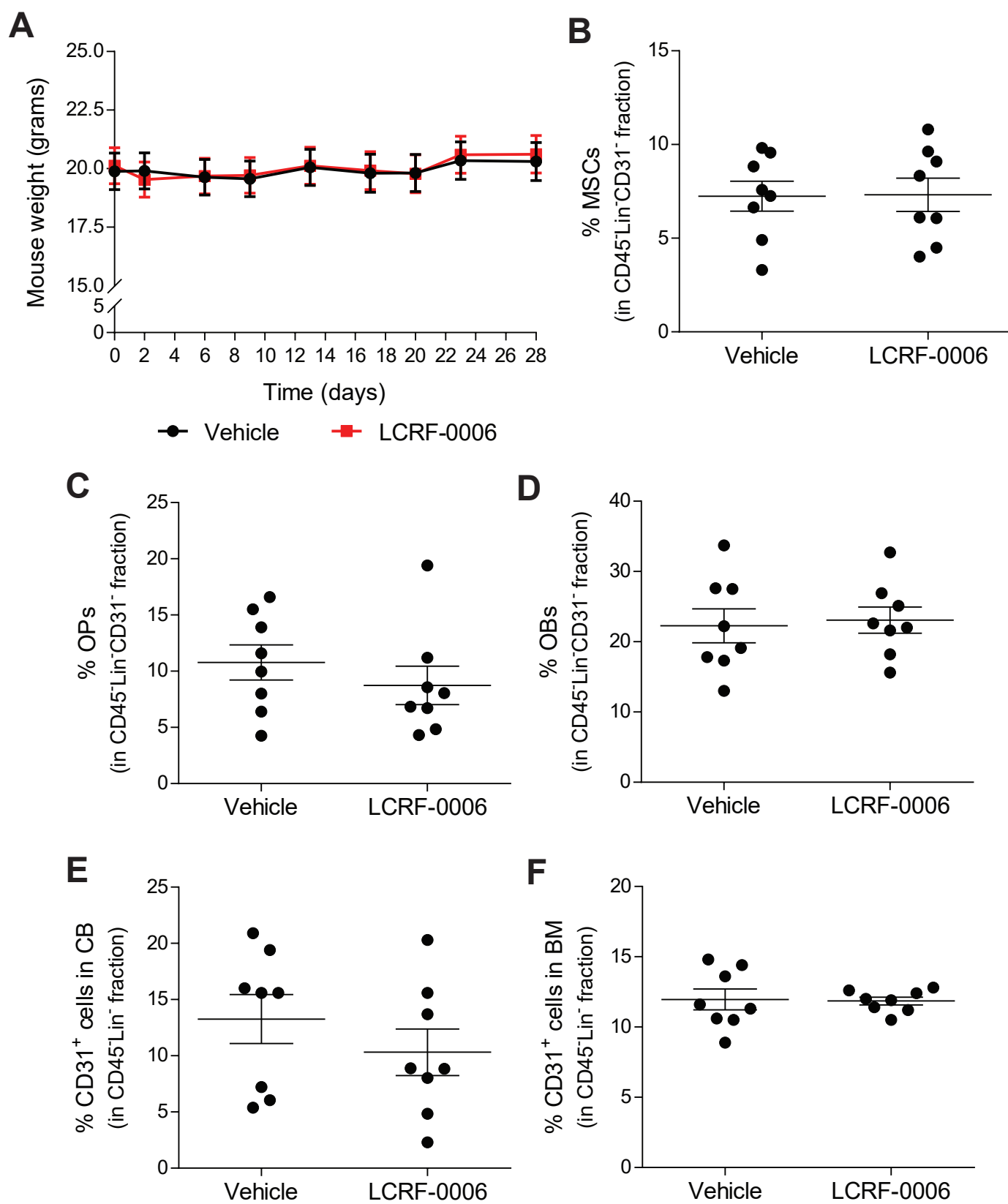

Supplement: Supplementary file 5 — Fig S5 [file FBA2-2-339-s005.pdf]

Supp. Fig. 6

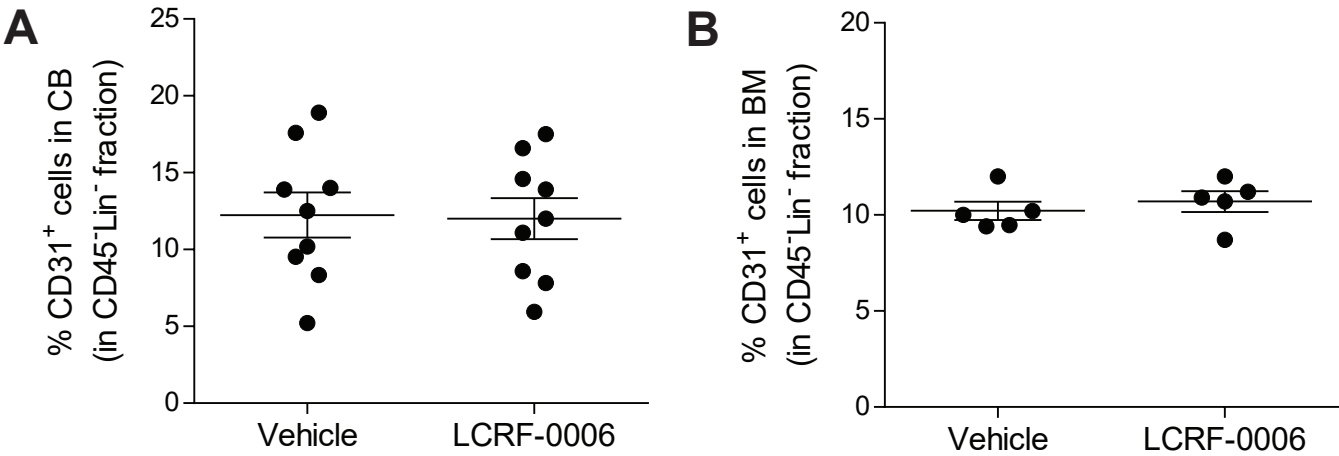

Supplement: Supplementary file 6 — Fig S6 [file FBA2-2-339-s006.pdf]
