# Supplementary material for: LCRF‐0006, a small molecule mimetic of the N‐cadherin antagonist peptide ADH‐1, synergistically increases multiple myeloma response to bortezomib
Source: FASEB Bioadv. 2020 Jun 15;2(6):339–53. doi: 10.1096/fba.2019-00073 (PMC7325588; doi:10.1096/fba.2019-00073)
Supplement: Supplementary file 7 — Table S1 [file FBA2-2-339-s007.pdf]

Supplemental Table 1. Complete blood counts of C57Bl/KaLwRij mice[1] following 28-day 100mg/kg/day LCRF-0006 treatment

Parameter	Treatment			p-value[3]
	Un-treated[2]	Vehicle[2]	LCRF-0006[2]	
	n =8 (5m & 3f)	n =8 (4m & 4f)	n =8 (4m & 4f)	
WBCs (K/µl)	5.92 ± 0.35	4.99 ± 0.57	5.49 ± 0.61	n.s.
Neutrophils (K/µl)	1.05 ± 0.18	0.68 ± 0.11	1.02 ± 0.18	n.s.
Lymphocytes (K/µl)	4.54 ± 0.18	4.05 ± 0.44	4.15 ± 0.37	n.s.
Monocytes (K/µl)	0.25 ± 0.04	0.18 ± 0.03	0.17 ± 0.04	n.s.
Eosinophils (K/µl)	0.07 ± 0.03	0.06 ± 0.02	0.11 ± 0.04	n.s.
Basophils (K/µl)	0.02 ± 0.01	0.02 ± 0.01	0.04 ± 0.01	n.s.
RBCs (M/µl)	7.59 ± 0.20	7.97 ± 0.10	7.83±0.13	n.s.
Hemoglobin (g/dl)	9.13 ± 0.14	9.55 ± 0.09	9.53 ± 0.14	n.s.
Hematocrit (%)	37.96 ± 0.99	39. 2± 0.39	39.0 ± 0.63	n.s.
MCV (fl)	50.08 ± 0.29	49.2 ± 0.17	49.8 ± 0.21	n.s.
MCH (pg)	12.05 ± 0.19	12.0 ± 0.13	12.2 ± 0.14	n.s.
MCHC (g/dl)	24.09 ± 0.44	24.4 ± 0.21	24.4 ± 0.33	n.s.
RDW (%)	16.41 ± 0.22	15.9 ± 0.20	16.9 ± 0.65	n.s.
Platelets (M/µl)	0.74 ± 0.06	1.05 ± 0.07	0.99 ± 0.09	n.s.
MPV (fl)	4.79 ± 0.16	4.95 ± 0.06	5.10 ± 0.15	n.s.

[1] 11-12 weeks of age at the time of cardiac bleed

[2] mean ± SEM

[3] Kruskal-Wallis test (with Dunn's multiple comparisons test)

WBCs (white blood cells), RBCs (red blood cells), MCV (mean corpuscular volume), MCH (mean corpuscular hemoglobin), MCHC (mean corpuscular hemoglobin concentration), RDW (red blood cell distribution width), MPV (mean platelet volume), n.s. (not significant)
